# Daptomycin-Induced Eosinophilic Pneumonia Mimicking Multifocal Pneumonia

**DOI:** 10.7759/cureus.30521

**Published:** 2022-10-20

**Authors:** Randa Abd Algayoum, Ahmed Elsherif, Zarak H Khan, George Roman

**Affiliations:** 1 Internal Medicine, St. Mary Mercy Hospital, Livonia, USA; 2 Endocrinology, Diabetes and Metabolism, University of Toledo, Toledo, USA

**Keywords:** acute eosinophilic pneumonia, daptomycin induced pneumonitis, bronchoalveolar lavage, transbronchial biopsy

## Abstract

A 71-year-old female presented to the emergency department with worsening dyspnea, dry cough, malaise, weight loss, fever, chills, and diaphoresis for one week. The patient had been hospitalized four weeks prior with right knee methicillin-resistant Staphylococcus aureus (MRSA) bursitis and was initially treated with IV vancomycin but was switched to IV daptomycin at the time of discharge for convenience of dosing. On presentation to the ED, vitals were normal. Physical examination revealed bilateral scattered rhonchi and crepitations. Chest X-ray revealed new patchy bilateral interstitial and airspace opacities concerning for multifocal pneumonia. Labs were pertinent for mild peripheral eosinophilia. CT chest revealed moderate diffuse ground glass opacities involving both lungs, with subpleural predominance and some areas of septal thickening seen as well. Daptomycin-induced pneumonitis was suspected, and empiric antibiotics were discontinued. The patient subsequently underwent fiberoptic bronchoscopy with bronchoalveolar lavage (BAL) and transbronchial lung biopsy. BAL fluid showed leukocytosis and eosinophilia of 25 mm^3^. Right upper lobe biopsy demonstrated foci of alveolar spaces with collections of eosinophils and histiocytes consistent with acute eosinophilic pneumonia. The patient was started on oral prednisone and albuterol breathing treatments with significant improvement after 48 hours from admission. She was discharged on albuterol inhalers and prednisone taper.

Acute eosinophilic pneumonia (AEP) is a lung condition that can be rapidly progressive, leading to significant morbidity and mortality. Daptomycin-induced AEP can mimic community-acquired pneumonia, resulting in delayed diagnosis and management. Recognizing the temporal association between drug initiation and the development of symptoms is crucial in the diagnosis of drug-induced AEP. If it is recognized and treated in a timely manner, the prognosis is generally excellent, with rapid and complete clinical recovery as demonstrated by our case.

## Introduction

Daptomycin (DAP) is a novel cyclic lipopeptide with bactericidal activity that was approved for use in 2003 by the United States (US) Food and Drug Administration (FDA) for treating methicillin-resistant Staphylococcus aureus. With the increasing use of daptomycin, a literature review revealed multiple case reports of drug-induced eosinophilic pneumonia [1]. Pulmonary eosinophilia was added to the adverse events, post-marketing experience section label for daptomycin in 2007 [1]. Eosinophilic pneumonia is a rare, but serious respiratory syndrome, which has been linked to several medications and chemicals. We present a case of daptomycin-induced eosinophilic pneumonia, mimicking multifocal pneumonia.

## Case presentation

**Figure 1:**
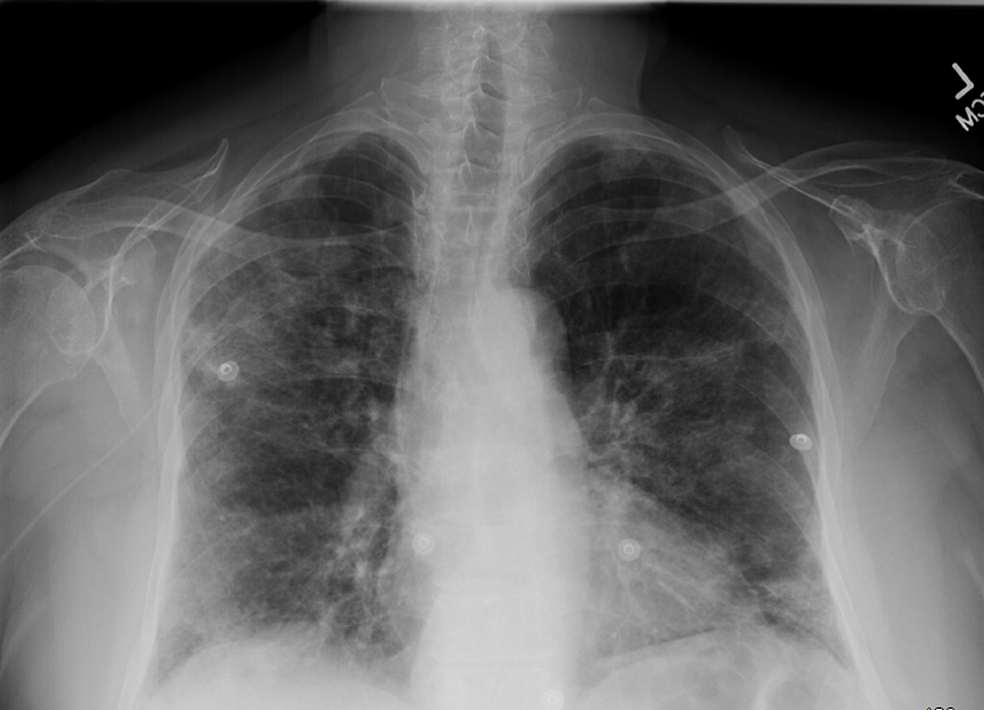
CXR demonstrating new patchy bilateral and airspace opacities concerning for multifocal pneumonia vs atypical infection

A 71-year-old female with no significant past medical history and no history of travel or ill contacts, presented to the emergency department with shortness of breath, dry cough, malaise, weight loss, fever, chills, and diaphoresis for one week, progressing to limiting her daily activities. The patient had been hospitalized four weeks prior to her presentation, with right knee methicillin-resistant Staphylococcus aureus (MRSA) bursitis and was initially treated with vancomycin and switched to daptomycin at the time of discharge for convenience of dosing, receiving a total of three weeks of antibiotic therapy. On admission, she appeared to be in respiratory distress. Physical examination demonstrated diffuse bilateral scattered expiratory rhonchi and crepitations. Extensive infectious workup, including influenza, Legionella, Histoplasma, Aspergillus, Mycoplasma, acid-fast bacillus (AFB), TB quantiferon, and sputum cultures, was unremarkable. The case was encountered before coronavirus disease 2019 (COVID-19), therefore it was not tested. Chest X-ray revealed bilateral opacities concerning for multifocal pneumonia vs atypical infection as demonstrated below in Figure 1. Labs were pertinent for mild peripheral eosinophilia. The patient was started on empiric IV antibiotics as well as nebulized bronchodilators and was admitted to the medical floor.

**Figure 2:**
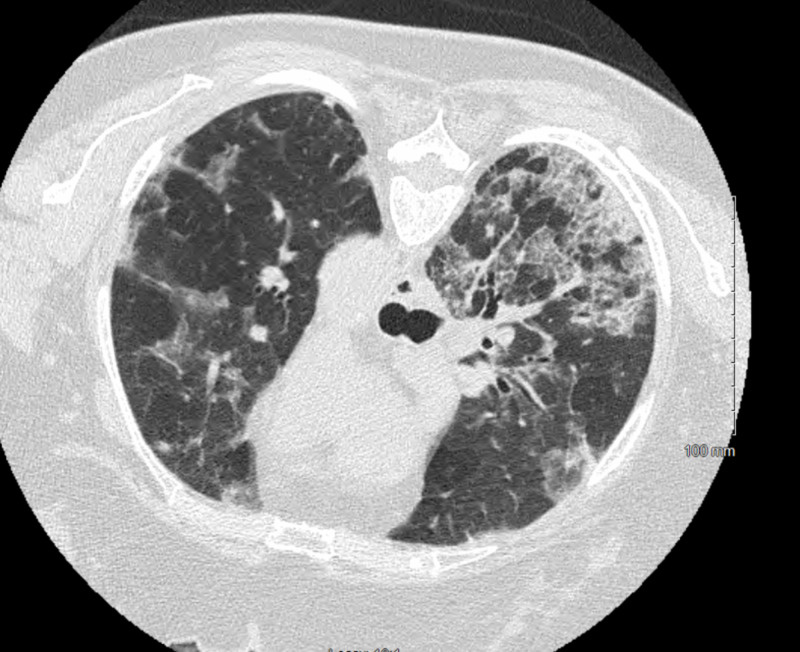
CT axial section demonstrating multifocal moderate geographic ground glass opacities bilaterally, distribution relatively diffuse with a peripheral predominance

**Figure 3:**
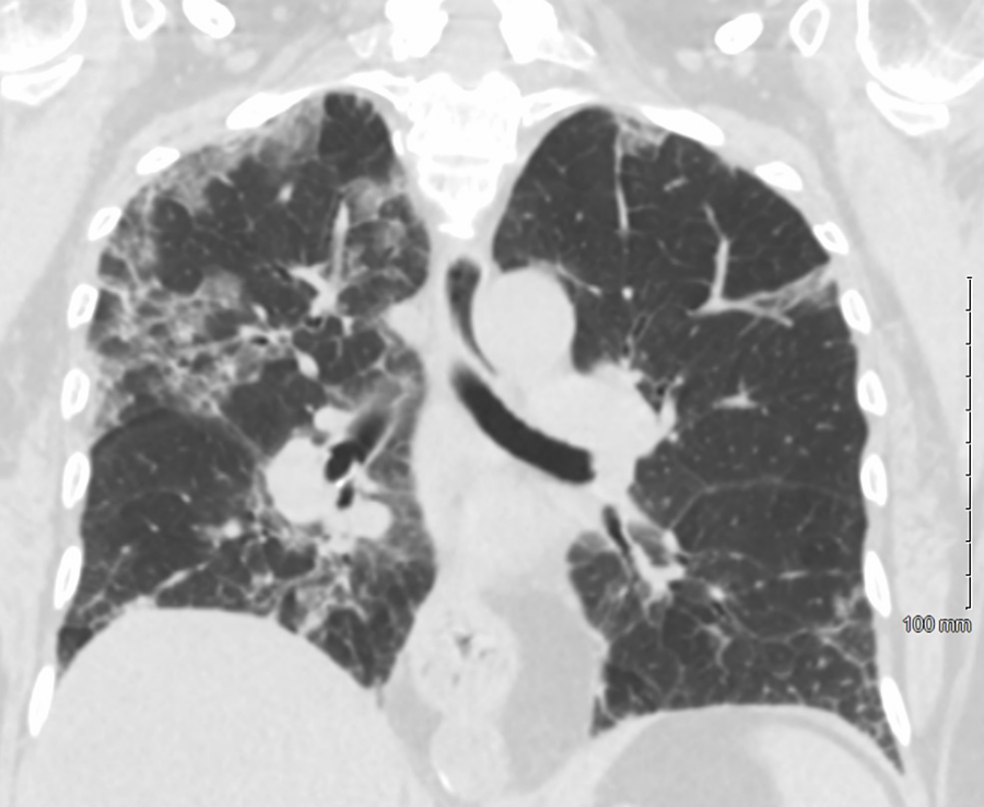
CT coronal lung window, redemonstrating diffuse bilateral ground glass opacities with peripheral predominance and some areas of septal thickening

The patient continued to desaturate, with increasing oxygen requirements escalating to non-invasive positive pressure ventilation. No significant improvement was noted within 48 hours. Pulmonology and Infectious disease services were consulted. CT chest without contrast was obtained for further evaluation and the findings are shown in Figures 2, 3.

Given her history, daptomycin-induced pneumonitis was suspected and antibiotics were immediately discontinued. The patient subsequently underwent fiberoptic bronchoscopy with bronchoalveolar lavage (BAL) and transbronchial lung biopsy. BAL fluid showed leukocytosis 385 mm^3^ and eosinophilia 25 mm^3^. Right upper lobe biopsy demonstrated foci of alveolar spaces with collections of eosinophils and histiocytes surrounded by thickened alveolar septae with focal organization and focal hyaline membrane formation. Findings were consistent with acute eosinophilic pneumonia (AEP). Final BAL fluid cultures were negative for bacterial and fungal growth. Histoplasma and legionella antigens were negative. The comprehensive viral panel was unremarkable. Daptomycin was discontinued, and the patient was started on oral prednisone with significant improvement noted after 48 hours. She was weaned off oxygen and discharged home on albuterol inhalers and prednisone taper. Outpatient follow-up within a few weeks, fortunately, showed complete recovery.

## Discussion

The exact mechanism of DEP is not yet well understood. One of the proposed mechanisms is thought to be due to daptomycin binding to pulmonary surfactant and accumulating in the alveoli causing injury to the tissues. The pathophysiology involves an antigen-mediated response, activating alveolar macrophages and T helper-2 cells, leading to the release of interleukin IL-5 and, therefore, more eosinophil production in the lungs [2].

AEP is diagnosed by the presence of febrile illness of less than 5 days duration, diffuse bilateral pulmonary infiltrates, hypoxemia (partial pressure of oxygen of less than 60 mmHg or pulse oximetry reading of <90% on room air), and BAL with greater than 25% eosinophils or eosinophilic pneumonia demonstrated by lung biopsy [2]. Solomon and Schwarz have put forth five criteria to diagnose drug-induced eosinophilic pneumonia, in addition to fulfilling the criteria mentioned above. It includes the presence of simple, acute, or chronic eosinophilic pneumonia by current diagnostic criteria, the presence of a likely or potential candidate drug or toxin in an appropriate time frame, no other causes of eosinophilic pneumonia (parasites, fungal infection), clinical improvement after cessation of the suspect drug or toxin, and recurrence of eosinophilic pneumonia with rechallenge to the drug or toxin. Forty cases of daptomycin-induced AEP were reported by Hirai et al., of whom 73% received a treatment dose of more than 6 mg/kg/day for a median of 14.8 days. The rest of the cases were treated with less than 6 mg/kg/day of daptomycin for a median of 23 days [3]. Some of the risk factors identified by Soldevila et al. for developing daptomycin-induced AEP include advanced age, high values of the Charlson comorbidity score, high total cumulative daptomycin dose, specifically more than 10 g, and prolonged therapy of more than two weeks [4].

The clinical presentation may vary from mild respiratory complaints to the minimal requirement of oxygen, up to acute respiratory distress syndrome (ARDS) requiring mechanical ventilation. Patients present with an acute illness of less than four weeks duration. Nonproductive cough, dyspnea, and fever are present in almost every patient. Associated symptoms can include malaise, myalgias, night sweats, chills, and pleuritic chest pain. Dyspnea was the most commonly reported symptom associated with eosinophilic pneumonia, followed by the presence of either pulmonary infiltrates or opacities on chest X-ray or CT [5]. Diagnosis mainly depends on a thorough clinical history, laboratory tests, and radiographic studies. The temporal association between exposure to the drug and clinical presentation is crucial in the diagnosis of drug-induced AEP. The chest X-ray may show only subtle reticular or ground glass opacities, often with Kerley B lines [6]. With progression, it can present with bilateral diffuse or reticular ground glass opacities. High-resolution computed tomography (HRCT) scans are not essential to the diagnosis but can help with guiding the selection of an area for BAL and further characterizing the opacities. HRCT is always abnormal in patients with AEP and is also characterized by bilateral, random, and patchy ground-glass or reticular opacities. Centrilobular nodules and air-space consolidation can be seen in approximately 50% and 40%, respectively. Bilateral pleural effusions are present in most patients [7].

Bronchoalveolar lavage is performed in the majority of the patients to quantitate eosinophilia and to exclude other etiologies. A differential cell count showing eosinophilia >25% and meeting the criteria discussed above is diagnostic. This threshold is noted to be diagnostic of eosinophilic interstitial lung disease in the American Thoracic Society Clinical Practice Guidelines [8].

Initial treatment usually includes supportive care with supplemental oxygen and mechanical ventilation, if required, along with systemic glucocorticoids and empiric antibiotics, which can be modified when culture results are available [9]. Although there is a lack of clinical trial data, most patients are seen to experience progressive respiratory failure in not treated with systemic glucocorticoids. However, improvement is rapid (within 12 to 48 hours) in response to intravenous or oral glucocorticoid therapy [7]. A commonly recommended regimen based on the literature available is 60-125 mg intravenous methylprednisolone every six hours in critically ill patients with respiratory failure, and oral prednisone 40-60 mg for less severe cases daily with tapering over two to six weeks depending on the resolution of symptoms [7].

## Conclusions

Eosinophilic pneumonia may lead to rapidly progressive respiratory failure, and a delayed diagnosis can lead to increased morbidity and mortality. Management includes discontinuing the drug and systemic corticosteroids, which have shown great outcomes and recovery. Symptoms and presentation may mimic pneumonia, therefore, clinicians should have a low threshold to recognize the symptoms in patients receiving daptomycin.
